# Validation of the Quick Dementia Rating System (QDRS): diagnostic properties and cutoff values

**DOI:** 10.1590/1980-5764-DN-2026-0520

**Published:** 2026-08-24

**Authors:** Joana Emilia Senger, Renata Kochhann, Daniel Arnold, Marco Antonio de Bastiani, Ariele Detogni, Neide Maria Bruscato, Emilio Hideyuki Moriguchi, João Senger, Anelise Vivian Fuhr, André Luiz Rodrigues Palmeira, Eduardo Rigon Zimmer, Luiz Antonio Nasi, Sheila Cristina Ouriques Martins, Wyllians Vendramini Borelli

**Affiliations:** 1 Hospital Moinhos de Vento, Centro da Memória, Porto Alegre RS, Brazil.; 2 Universidade Federal do Rio Grande do Sul, Programa de Pós-Graduação em Farmacologia e Terapêutica, Porto Alegre RS, Brazil.; 3 Instituto Moriguchi, Veranópolis RS, Brazil.; 4 Universidade Federal do Rio Grande do Sul, Programa de Pós-Graduação em Ciências da Saúde, Porto Alegre RS, Brazil.; 5 Hospital de Clínicas de Porto Alegre, Porto Alegre RS, Brazil.; 6 Universidade Federal do Rio Grande do Sul, Departamento de Farmacologia, Porto Alegre RS, Brazil.; 7 Universidade Federal do Rio Grande do Sul, Programa de Pós-Graduação em Bioquímica, Porto Alegre RS, Brazil.; 8 Hospital Moinhos de Vento, Unidade de Neurociência, Porto Alegre RS, Brazil.; 9 Hospital Moinhos de Vento, Porto Alegre RS, Brazil; 10 Universidade Federal do Rio Grande do Sul, Departamento de Ciências Morfológicas, Porto Alegre RS, Brazil.

**Keywords:** Dementia, Accuracy, Mental Status and Dementia Tests, Validation Study, Demência, Acurácia Diagnóstica, Testes de Estado Mental e Demência, Estudo de Validação

## Abstract

Dementia underdiagnosis persists because lengthy staging tools hinder routine clinical use. The Quick Dementia Rating System (QDRS) offers a brief, informant-based alternative.

**Objective:**

This study validates the Brazilian Portuguese QDRS and establishes optimal cutoff values for diagnostic accuracy.

**Methods:**

Individuals from primary care and a secondary memory clinic in Southern Brazil were included. Informants completed the QDRS prior to clinical evaluation. All participants underwent comprehensive neurological and neuropsychological assessment, including the Clinical Dementia Rating (CDR), Mini-Mental State Examination (MMSE), and Montreal Cognitive Assessment (MoCa). Psychometric properties (internal consistency and concurrent validity), diagnostic accuracy, and staging agreement between different diagnostic groups and CDR categories were investigated.

**Results:**

A total of 180 participants were included in this study (59 cognitively unimpaired, 63 with mild cognitive impairment [MCI], and 58 with dementia). The QDRS demonstrated excellent internal consistency (Cronbach’s α=0.94) and strong concurrent validity with the CDR-Sum of Boxes (p=0.82, p<0.001), MMSE and MoCa scores. Agreement between corresponding QDRS and CDR domains ranged from moderate to good (Intraclass Correlation Coefficient — ICC=0.73–0.89). Diagnostic accuracy for dementia was excellent relative to both clinical diagnosis (cutoff 3.25; Area Under the Curve — AUC=0.98) and CDR staging (cutoff 4.25; AUC=0.97), with sensitivities and specificities exceeding 92%. Staging agreement with the CDR was substantial (weighted Kappa=0.64).

**Conclusion:**

The Brazilian Portuguese QDRS is a reliable and efficient instrument for dementia screening and staging. Large-scale testing in clinical practice and population-based research are warranted.

## RESUMO.

O subdiagnóstico de demência persiste porque ferramentas de estadiamento longas dificultam o uso clínico rotineiro. O Quick Dementia Rating System (QDRS) oferece uma alternativa breve, baseada em informantes.

**Objetivo:**

Validar a versão em português brasileiro do QDRS e estabelecer valores de corte ideais para a acurácia diagnóstica.

**Métodos:**

Foram incluídos indivíduos da atenção primária e de uma clínica de memória no Sul do Brasil. Os informantes preencheram o QDRS antes da avaliação clínica. Todos os participantes foram submetidos a uma avaliação neurológica e neuropsicológica, incluindo o Clinical Dementia Rating (CDR), o Miniexame do Estado Mental (MEEM) e o Montreal Cognitive Assessment (MoCA). Foram investigadas propriedades psicométricas (consistência interna e validade concorrente), acurácia diagnóstica e concordância de estadiamento entre diferentes grupos diagnósticos e categorias do CDR.

**Resultados:**

O total de 180 participantes foi incluído neste estudo (59 cognitivamente saudáveis, 63 com comprometimento cognitivo leve [CCL] e 58 com demência). O QDRS demonstrou excelente consistência interna (α de Cronbach=0,94) e forte validade concorrente com os escores do CDR-Sum of Boxes (p=0,82, p<0,001), MEEM e MoCA. A concordância entre os domínios correspondentes do QDRS e do CDR variou de moderada a boa (coeficiente de correlação intraclasse — CCI=0,73–0,89). A acurácia diagnóstica para demência foi excelente em relação tanto ao diagnóstico clínico (ponto de corte 3,25; AUC=0,98) quanto ao estadiamento pelo CDR (ponto de corte 4,25; AUC=0,97), com sensibilidades e especificidades superiores a 92%. A concordância de estadiamento com o CDR foi substancial (Kappa ponderado=0,64).

**Conclusão:**

O QDRS em português brasileiro é um instrumento confiável e eficiente para o rastreio e estadiamento de demência. Testes em larga escala na prática clínica e pesquisas de base populacional são necessários.

## INTRODUCTION

 Dementia remains a significant global public health challenge, comprehending high rates of underdiagnosis^1^. Dementia underdiagnosis is driven by a complex interplay of factors, including limited public awareness, the time-consuming diagnostic protocols, and lack of scalable screening tools^2^. To mitigate dementia underdiagnosis, health workers must prioritize the use of high-precision instruments capable of stratifying cognitive decline within community settings and specialized clinics^3^. 

 The Clinical Dementia Rating (CDR) is currently considered the gold standard instrument for staging of cognitive impairment^4^. It is a clinician-administered, semi-structured interview widely used to reliably classify the severity of cognitive impairment and its impact on daily activities^5,6^. By integrating both patient and informant data, the CDR remains relatively independent of age and education, demonstrating high consistency and validity across clinical and research settings^7^. However, the CDR is time-intensive and requires specialized training, which limits its scalability in routine clinical practice and large-scale population studies. 

 The Quick Dementia Rating System (QDRS) is a brief, 5-minute assessment tool designed for completion by either patients or informants^8^. The QDRS aligns closely with the domains evaluated in the CDR, and provides a global measure of disease severity with high accuracy in distinguishing dementia stages, specially considering that it is a CDR-boxes transformed into a self- or informant-administered staging tool^8^. Its streamlined format ensures rapid administration and high reliability, making it an excellent choice for routine clinical practice, large-scale screening, and resource-constrained research settings^8,9^. Previous studies have demonstrated a robust correlation between the QDRS and the CDR across diverse clinical and cultural contexts^10,11^. Major differences between the QDRS and CDR include the scoring system (CDR has six domains, while QDRS has ten), time required (CDR is 60-min long, while the QDRS is 5-min long), training requirements (CDR requires training, while QDRS does not), and administration (CDR is semi-structured by a trained clinician, while the QDRS is self-administered or informant-administered). 

 In Brazil, substantial linguistic and cultural variation across regions, and a high burden of underdiagnosed cognitive disorders within the public healthcare system, pose challenges to the validity and applicability of screening instruments. Therefore, the aim of this study is to evaluate the diagnostic properties of the QDRS in Brazilian Portuguese, and to provide optimal cutoff values for distinguishing different diagnostic groups. 

## METHODS

### Development of the Quick Dementia Rating System

 The QDRS was originally developed and validated in a dementia specialty practice^8^. It consists of ten domains: the six original categories found in the CDR (memory, orientation, judgment and problem solving, community affairs, home and hobbies, and personal care), along with four additional areas: language, mood, behavior, and attention. Each domain is rated on a Likert scale with scores of 0, 0.5, 1, 2, and 3. The total score is calculated by summing the points across all domains, ranging from 0 to 30, with higher scores indicating greater cognitive and functional impairment. This structure allows the QDRS to capture both cognitive and behavioral components^8^. 

### Translation procedures and administration of the Quick Dementia Rating System

 The cross-cultural adaptation and translation of the QDRS into Brazilian Portuguese were conducted in accordance with international guidelines^12^, and are presented in Supplementary Methods and Table S1 (Supplementary Material available at https://www.demneuropsy.org/wp-content/uploads/2026/06/DN-2026.0520-Supplementary-Material.docx). With permission from the original author, two bilingual translators independently translated the instrument from English to Brazilian Portuguese. These versions were reviewed by a professional translator and the corresponding author to produce a synthesized version. Any discrepancies or ambiguities were resolved through discussion and consensus among the translation team. 

 A professional English teacher and native speaker without a background in cognition was asked to back-translate the QDRS into English. The expert translator examined both the back-translated and original English versions to guarantee consistency and equivalency. An initial evaluation with ten healthcare professionals and ten patients was conducted to qualitatively evaluate the adequacy of the tool. No changes were made at this point, and the instrument was then considered adequate for application. 

 In both primary and secondary settings, the informant-based QDRS was completed independently by the informants, according to their perceptions of the patient. To ensure the autonomy of the informant’s report, the professional team did not interfere with the responses, although they remained available to clarify any doubts after completion. Informants were mandatory, and were required to exchange during 10 or more hours per week of patient contact. Each questionnaire was completed by a single informant. 

### Study participants

 A total of 180 individuals were included in this study, from two independent cohorts in Southern Brazil. This sample encompassed both community-dwelling participants in primary care (*Estudo Longitudinal da Cognição de Idosos de Veranópolis* — ELVE), and patients from a specialized secondary memory clinic (*Porto Alegre Longitudinal Study of Aging and Degenerative diseases* — PORTO-AD), irrespective of age and sex (Supplementary Table S2). To ensure diagnostic consistency, all participants were evaluated between September 2024 and January 2026 by a single transdisciplinary team comprising a neurologist, a nurse, and a neuropsychologist. All individuals across the cognitive spectrum were eligible for inclusion. Exclusion criteria consisted of psychiatric comorbidities, stroke, or severe sensory deficits, including severe auditory or visual loss. All participants consented to participate in this study according to the study protocol numbers (ELVE: 8.127.531; PORTO-AD: 6.308.735). 

### Medical assessment

 Participants were classified by a trained neurologist based on the International Working Group diagnostic criteria^13^, considering that all dementia cases were identified as amnestic-type AD following Brazilian guidelines (36533157). Dementia cases were investigated in each setting (community or specialized center) and followed the Brazilian guidelines, presenting no laboratory exams associated with cognitive decline, and neuroimaging findings without diagnostic criteria for mixed-type dementia or vascular dementia. They were divided into three diagnostic groups: cognitively unimpaired (CU), mild cognitive impairment (MCI) and dementia. Regarding the study procedures, the QDRS was completed by the informant prior to the clinical encounter. During the subsequent consultation, each patient-informant underwent a comprehensive assessment, which included the CDR^14^, a full neuropsychological battery, and a formal clinical examination. 

### Neuropsychological evaluation

 Each patient underwent a cognitive battery during the visit to determine their clinical status. Global cognition was screened using the Mini-Mental State Examination (MMSE) and the Montreal Cognitive Assessment (MoCA), while executive and language functions were assessed via Verbal Fluency tasks (semantic and phonemic) and Clock Drawing Test (CDT). These assessments were administered by a neuropsychologist or a trained nurse. Additionally, a neurologist or neuropsychologist conducted independent, semi-structured interviews with both the patient and a collateral informant. A complete neuropsychological battery was not available for all participants; all individuals underwent a complete CDR interview to stage cognitive impairment severity, and usually took 45 to 60-minutes^14^. The CDR is a widely accepted tool to identify MCI^6,15^. Global CDR scores were interpreted as follows: 0 for CU; 0.5 for MCI or very mild dementia; and 1, 2, or 3 for mild, moderate, and severe dementia, respectively. 

### Statistical analysis

 Data normality for continuous variables was assessed using the Shapiro-Wilk test. Descriptive statistics are presented as mean ± standard deviation (SD) for normally distributed data, while categorical variables are reported as absolute frequencies and percentages (%). A correlation matrix was generated to evaluate the relationship between quantitative variables (Supplementary Figure S1). The psychometric properties of the QDRS were assessed through internal consistency (Cronbach’s alpha) and concurrent validity using Spearman’s rank correlation with gold-standard measures (CDR, MMSE, MoCA). Additionally, the agreement between individual QDRS domains and their corresponding CDR domains (e.g., QDRS-Memory vs. CDR-Memory) was assessed using the Intraclass Correlation Coefficient (ICC), specifically a two-way mixed model looking for consistency. 

 Diagnostic performance was evaluated via Receiver Operating Characteristic (ROC) curve analyses, calculating the Area Under the Curve (AUC) and optimal cut-off scores using the Youden Index for both clinical diagnosis and CDR staging. Finally, staging agreement between QDRS-derived clinical stages and the global CDR score was assessed using linear weighted Cohen’s Kappa. Sensitivity analyses were conducted to evaluate diagnostic performance of QDRS using two subcohorts: one with community-dwelling participants (n=75) and another with secondary care participants (n=105). Only non-null data was used. The significance level was set at p<0.05 for all analyses. Statistical analyses were performed using R (version 4.4.1). 

## RESULTS

### Sample characteristics

 A total of 180 individuals were included in this study (ELVE-cohort, n=75; PORTO-AD cohort, n=105, Supplementary Figure S4). The mean age was 69.4 ± 10.8 years (range 38-89), with a mean education of 11.1±6.9 years (range 1–38). The mean MMSE score was 23.2±5.3. Regarding ethnic composition, the sample was composed mostly by White individuals (92%, Table 1). The cohort was divided into three groups: 59 CU, 63 with MCI, and 58 with dementia. Informants included adult children (38.89%), spouses (34.44%), caregivers (4.44%), and others (22.22%). Sample characteristics and QDRS scores, stratified by diagnosis and stage, are presented in Table 1. 

**Table 1:** Demographic and clinical characteristics by clinical diagnostic groups.

		Total (n=180)	CU n=59	MCI n=63	Dementia n=58	p-values
Age, mean years (SD)		69.4 (10.8)	62.1 (10.3)^*,†^	70.3 (10.2)^‡^	75.9 (6.7)	**<0.001**
Education, mean years (SD)		11.1 (6.9)	14.0 (7.2)^*,†^	9.0 (5.6)^‡^	10.4 (7.1)	**<0.001**
Female Sex, n (%)		113 (63%)	48 (81%)*	28 (44%)^‡^	37 (64%)	**<0.001**
Race, n (%)						
	White	166 (92%)	50 (85%)	60 (95%)	56 (97%)	0.086
	Non-White	14 (7.9%)	9 (15.4%)	3 (4.8%)	2 (3.4%)	
MMSE score, mean (SD)		23.2 (5.3)	26.9 (2.4)^*,†^	24.4 (3.3)^‡^	18.1 (5.3)	**<0.001**
MoCA score, mean (SD)		19.2 (6.2)	24.2 (3.6)^*,†^	19.9 (4.0)^‡^	13.2 (5.3)	**<0.001**
Semantic Fluency, mean (SD)		9.6 (4.9)	12.3 (4.9)^*,†^	9.7 (4.5)^‡^	6.7 (3.7)	**<0.001**
CDR Global Score, n (%)						
	0	71 (39%)	54 (92%)^*,†^	17 (27%)^‡^	0 (0%)	**<0.001**
	0.5	53 (29%)	5 (8.5%)^*,†^	43 (68%)^‡^	5 (8.6%)	
	1	34 (19%)	0 (0%)^*,†^	3 (4.8%)^‡^	31 (53%)	
	2	20 (11%)	0 (0%)^*,†^	0 (0%)^‡^	20 (34%)	
	3	2 (1.1%)	0 (0%)^*,†^	0 (0%)^‡^	2 (3.4%)	
CDR Sum of Boxes, mean (SD)		2.6 (3.9)	0.1 (0.2)	1.0 (1.3)^‡^	7.0 (4.2)	**<0.001**
QDRS Total Score, mean (SD)		4.9 (6.2)	0.7 (1.6)^*,†^	2.9 (3.8)^‡^	11.1 (6.5)	**<0.001**
QDRS Cognitive Subscale, mean (SD)		2.1 (2.8)	0.2 (0.6)^*,†^	1.1 (1.6)^‡^	5.0 (2.9)	**<0.001**
QDRS Behavioral Subscale, mean (SD)		2.8 (3.6)	0.6 (1.2)*	1.8 (2.5)^‡^	6.1 (3.9)	**<0.001**

Abbreviations: SD, standard deviation; CU, Cognitively Unimpaired; MCI, Mild Cognitive Impairment; CDR, Clinical Dementia Rating; QDRS, Quick Dementia Rating System.

Notes: Pairwise comparison identifies p<0.05, where *CU vs. MCI; †CU vs. Dementia; ‡MCI vs. Dementia. Bold: when p < 0.05.

**Figure 1:**
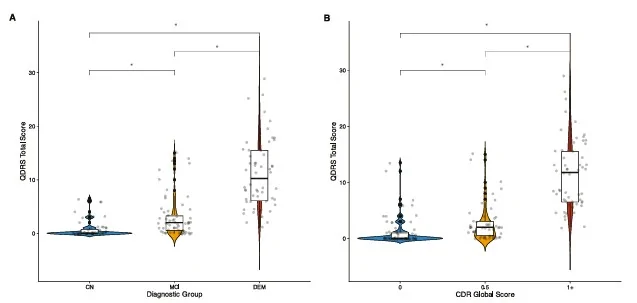
Quick Dementia Rating System total scores according to (A) clinical diagnostic groups (B) and Clinical Dementia Rating global score. Abbreviations: QDRS, Quick Dementia Rating System; CU, Cognitively Unimpaired; MCI, Mild Cognitive Impairment; DEM, Dementia. Significant differences were observed across groups (Kruskal-Wallis, p<0.001), with significant post-hoc differences for all pairwise comparisons.

 QDRS Total scores differed significantly across diagnostic groups (Figure 1A) and CDR scores (Figure 1B, p<0.001). Post-hoc analyses revealed significant differences between all pairs, in both gold-standard tools. 

### Internal consistency analysis

 The QDRS demonstrated high internal consistency. The Cronbach’s alpha for the QDRS total scale was 0.94, with subdomain reliability ranging from 0.89 for the Cognitive Subscal, to 0.91 for the QDRS Behavioral Subscale. The average inter-item correlation was 0.63, indicating strong cohesion among the scale’s domains while maintaining the ability to capture distinct aspects of impairment. 

### Concurrent validity

**Figure 2:**
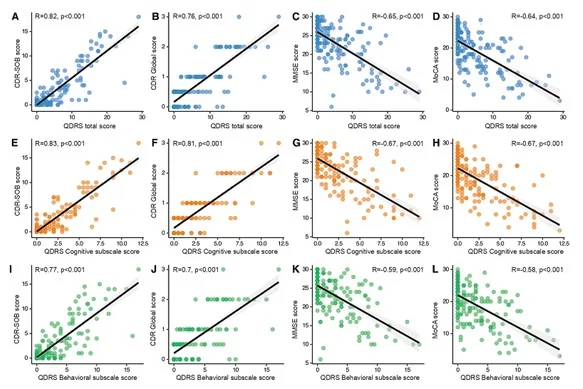
Concurrent validity of the Quick Dementia Rating System. Scatter plots showing Spearman correlations between Quick Dementia Rating System total and subscale scores and Clinical Dementia Rating-Sum of Boxes, Clinical Dementia Rating, Mini-Mental State Examination, and Montreal Cognitive Assessment scores. Quick Dementia Rating System scores increased progressively across Clinical Dementia Rating global stages, supporting strong concurrent validity. Abbreviations: CDR, Clinical Dementia Rating; MMSE, Mini-Mental State Examination; MoCa, Montreal Cognitive Assessment; CDR, Clinical Dementia Rating; SOB, Sum of Boxes; QDRS, Quick Dementia Rating System.

 The QDRS demonstrated strong concurrent validity when compared to the gold-standard CDR. There was a very strong correlation between the QDRS Total Score and the CDR Sum of Boxes (Spearman’s rho=0.82, p<0.001, Figure 2A), indicating high agreement between the informant-based QDRS and the clinician-based assessment. Spearman’s correlation coefficient between QDRS total scores and CDR, MMSE and MoCA scores were strong or very strong (Figure 2A-D). The correlation between QDRS Cognitive Subscale and CDR-Sum of Boxes (CDR-SOB), CDR, MMSE and MoCA scores varied from strong to very strong (Figure 2E-H). The correlation between QDRS Behavioral Subscale and CDR-SOB, CDR, MMSE and MoCA scores varied from moderate to strong (Figure 2I-L). Furthermore, QDRS scores progressively increased across CDR global stages (Figure 1). 

 To assess validity at the domain level, we calculated the ICC between corresponding items of the QDRS and CDR (Table 2). There was a good agreement for Memory, Judgement & Problem Solving, and Personal Care, while moderate agreement was found for Orientation, Community Affairs and Home & Hobbies. 

**Table 2:** Quick Dementia Rating System domains and their Intraclass Correlation Coefficient with matching Clinical Dementia Rating scoring items.

Domain/Score	ICC (95%CI)	Agreement	p-value
Memory	0.89 (0.86–0.92)	Good	<0.001
Orientation	0.79 (0.72–0.84)	Moderate	<0.001
Judgement & Problem Solving	0.84 (0.78–0.88)	Good	<0.001
Community Affairs	0.73 (0.62–0.80)	Moderate	<0.001
Home & Hobbies	0.73 (0.62–0.81)	Moderate	<0.001
Personal Care	0.82 (0.76–0.86)	Good	<0.001

Abbreviations: ICC, Intraclass Correlation Coefficient; CI, confidence interval.

### Operating characteristics

**Figure 3:**
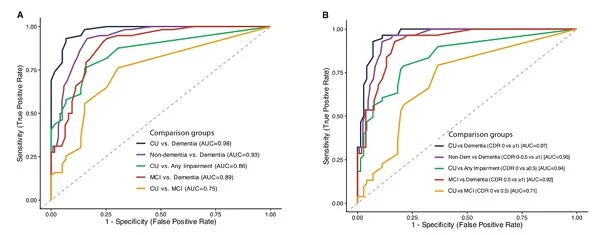
Diagnostic accuracy of the Quick Dementia Rating System. Receiver Operating Characteristic curves demonstrating the discriminative ability of the QDRS total score against (A) clinical diagnostic groups and (B) Clinical Dementia Rating stages. Curves illustrate the test’s ability to distinguish between different clinical scenarios. Abbreviations: CU, cognitively unimpaired; AUC, Area Under the Curve; MCI, mild cognitive impairment.

 To demonstrate the diagnostic accuracy of the QDRS, we compared it with the clinical diagnosis and CDR scores. Using clinical diagnosis as gold standard (Figure 3A), the QDRS exhibited very high diagnostic accuracy in distinguishing non-demented participants (MCI and CU) from dementia patients, with an AUC-ROC=0.98 (9% confidence interval — 95%CI [0.96–1], Table 3). The optimal cut-off score to detect dementia was >3.25, which yielded a sensitivity of 93.1% and a specificity of 93.2%, presenting a Youden index of 86.3 (Table 3). Considering the CDR as gold standard (Figure 3B), the QDRS also demonstrated very high diagnostic accuracy in distinguishing patients with CDR 1, 2 and 3 from CDR 0 and 0.5, with an AUC-ROC=0.97 (95%CI [0.94–1], Table 3). Considering the CDR, the optimal cut-off score to detect dementia was >4.25, which yielded a sensitivity of 92.9% and a specificity of 93%, presenting a Youden index of 85.9 (Table 3). 

**Table 3:** Areas Under the Curve for clinical diagnostic groups or Clinical Dementia Rating categories using Quick Dementia Rating System Total Scores, Quick Dementia Rating System Cognitive Subscale or Quick Dementia Rating System Behavioral Subscale.

		QDRS Total scores	QDRS Cognitive subscale	QDRS Behavioral subscale
Clinical diagnosis				
	CU vs. Any Impairment	0.86 (0.81–0.91)	0.86 (0.81–0.91)	0.82 (0.76–0.88)
	Non-Dementia vs. Dementia	0.93 (0.9–0.97)	0.94 (0.91–0.98)	0.9 (0.86–0.95)
	CU vs. MCI	0.75 (0.66–0.83)	0.75 (0.68–0.83)	0.7 (0.61–0.78)
	MCI vs. Dementia	0.89 (0.84–0.95)	0.91 (0.85–0.96)	0.85 (0.79–0.92)
	CU vs. Dementia	0.98 (0.96–1)	0.98 (0.96–1)	0.96 (0.93–0.99)
Clinical Dementia Rating stages				
	0 vs. ≥0.5	0.84 (0.79–0.9)	0.86 (0.81–0.92)	0.79 (0.73–0.86)
	0/0.5 vs. ≥1	0.95 (0.92–0.98)	0.96 (0.93–0.98)	0.92 (0.88–0.96)
	0 vs. 0.5	0.71 (0.62–0.8)	0.75 (0.66–0.83)	0.64 (0.55–0.73)
	0.5 vs. ≥1	0.92 (0.87–0.97)	0.93 (0.88–0.97)	0.9 (0.84–0.96)
	0 vs. ≥1	0.97 (0.94–1)	0.98 (0.96–1)	0.94 (0.9–0.98)

Abbreviations: QDRS, Quick Dementia Rating System; CU, Cognitively Unimpaired; MCI, Mild Cognitive Impairment.

 When distinguishing clinical diagnosis of CU from MCI, the QDRS showed good discriminative ability, with an AUC of 0.75 (95%CI [0.66–0.83], Table 3). A cut-off score of > 0.25 provided the best balance between metrics, resulting in a sensitivity of 76.2% and specificity of 69.5%, presenting a Youden index of 45.7 (Table 4). In distinguishing CDR 0 from 0.5, the QDRS showed good discriminative ability, with an AUC of 0.71 (95%CI [0.62–0.8], Table 3). A cut-off score of >0.25 provided the best balance between metrics, resulting in a sensitivity of 79.2% and specificity of 63.4%, presenting a Youden index of 42.6 (Table 4). Sensitivity analyses demonstrated very similar performance in distinguishing diagnostic groups and CDR stages in primary care (Supplementary Figure S2) and secondary care (Supplementary Figure S3). 

**Table 4:** Diagnostic properties in distinguishing clinical diagnostic groups or Clinical Dementia Rating categories using Quick Dementia Rating System Total Scores.

		AUC (95%CI)	Optimal cutoff	Sensitivity	Specificity	PPV	NPV	LR+	LR-
Clinical diagnosis									
	CU vs. Any Impairment	0.86 (0.81–0.91)	1.25	76	84.7	91.1	63.3	4.98	0.28
	Non-Dementia vs. Dementia	0.93 (0.9–0.97)	3.25	93.1	83.6	73	96.2	5.68	0.08
	CU vs. MCI	0.75 (0.66–0.83)	0.25	76.2	69.5	72.7	73.2	2.5	0.34
	MCI vs. Dementia	0.89 (0.84–0.95)	3.25	93.1	74.6	77.1	92.2	3.67	0.09
	CU vs. Dementia	0.98 (0.96–1)	3.25	93.1	93.2	93.1	93.2	13.73	0.07
Clinical Dementia Rating stages				
	0 vs. ≥0.5	0.84 (0.79–0.9)	1.25	78.9	78.9	85.1	70.9	3.73	0.27
	0 or 0.5 vs. ≥1	0.95 (0.92–0.98)	4.25	92.9	88.7	78.8	96.5	8.22	0.08
	0 vs. 0.5	0.71 (0.62–0.8)	0.25	79.2	63.4	61.8	80.4	2.16	0.33
	0.5 vs. ≥1	0.92 (0.87–0.97)	4.25	92.9	83	85.2	91.7	5.47	0.09
	0 vs. ≥1	0.97 (0.94–1)	4.25	92.9	93	91.2	94.3	13.19	0.08

Abbreviations: AUC, Area Under the Curve; CI, confidence interval; PPV, Positive Predictive Value; NPV, Negative Predictive Value; LR+, Positive Likelihood Ratio; LR, Negative Likelihood Ratio; CU, Cognitively Unimpaired; MCI, Mild Cognitive Impairment.

 To evaluate the clinical utility of the QDRS as a screening tool, we tested its diagnostic accuracy in distinguishing CU from any impairment, yielding similar accuracy values when compared with clinical diagnosis (AUC 0.86 [95%CI 0.81–0.91]) and CDR (AUC 0.84 [95%CI 0.79–0.9]). The same optimal cutoff value of >1.25 was calculated for this purpose using both gold standards, but slightly different sensitivity and specificity were demonstrated (Table 4). At the optimal cut-off, the QDRS demonstrated an LR+ above 10, indicating a large increase in the likelihood of dementia for positive test results. Conversely, the LR- below 0.10 suggests that a negative QDRS score effectively rules out significant cognitive impairment (Table 4). 

### Staging agreement

**Figure 4:**
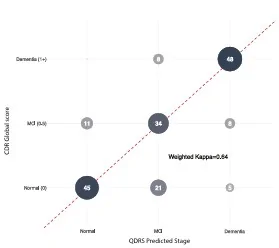
Bubble plot showing agreement between Quick Dementia Rating System-predicted clinical diagnostic groups and Clinical Dementia Rating stages. Abbreviations: CDR, Clinical Dementia Rating; MCI, mild cognitive impairment; QDRS, Quick Dementia Rating System.

 To assess the utility of the QDRS in determining disease severity, total scores were converted into clinical stages (CU, MCI, dementia) using aforementioned proposed cutoffs (0.5 and 4.5). The linear weighted Cohen’s Kappa was 0.64 (95%CI [0.55–0.73]), indicating strong concordance between the two instruments. The majority of participants fell along the diagonal (Figure 4), meaning the QDRS correctly classified the severity stage in 70.5% of cases. Disagreements were largely restricted to adjacent stages (e.g., classifying a CDR 0.5 as QDRS 0 or 1). 

## DISCUSSION

 This study validates the QDRS in Brazilian Portuguese, demonstrating its diagnostic performance as a brief and reliable alternative to the gold-standard CDR. The QDRS proved highly accurate in differentiating CU individuals from those with dementia, as well as distinguishing MCI from dementia, demonstrated by its excellent discriminatory power. These findings highlight the potential of the QDRS to streamline diagnostic workflows in both clinical and research settings, enabling rapid and reliable dementia staging while maintaining alignment with internationally recognized neuropsychological standards. 

 In terms of diagnostic accuracy, the QDRS demonstrated outstanding performance within Brazilian samples in distinguishing different diagnostic groups. The instrument effectively distinguished CU and MCI individuals from those with dementia, yielding exceptionally high AUC values. These findings closely replicate those reported in the original validation study^8^, and are consistent with the instrument’s robust performance across other global samples. To date, the QDRS has been successfully validated in multiple languages, including English^8^, Korean^11^ and Chinese^16^, and implemented in diverse settings. Across these studies, the QDRS has consistently demonstrated high diagnostic accuracy and robust discriminatory power in identifying individuals with dementia, which corroborates its reliability across diverse linguistic and cultural contexts. 

 The QDRS demonstrated high discriminative performance across different diagnostic categories. The comparable discriminative capacity observed in our sample for differentiating CU individuals from those with dementia further reinforces the cross-cultural validity of the QDRS as a globally applicable dementia staging instrument^10,11,16^. Paralleling the CDR scale, the QDRS is primarily designed to document the presence and severity of cognitive impairment rather than to establish specific etiological diagnoses^17^. A central advantage of this instrument lies in its operational efficiency, characterized by minimal training requirements and seamless integration into time-limited clinical workflows. Importantly, one of the main advantages in using the QDRS is its brevity, taking approximately 3–5 minutes for completion and easy correction^8^. Accordingly, the Brazilian Portuguese version of the QDRS represents a valuable tool for the identification and staging of cognitive impairment, with broad utility in clinical practice, clinical trials, prevention studies, community-based surveys, and biomarker research. 

 Regarding the screening of early functional loss, the QDRS demonstrated robust discriminative capacity in differentiating MCI from dementia, with an AUC of 0.89. However, its accuracy was moderate when distinguishing CU individuals from those with MCI, presenting an acceptable diagnostic property with an AUC of 0.75^10^. This discrepancy mirrors the inherent complexity of identifying MCI, which represents a transitional and often heterogeneous stage of cognitive impairment^18,19^. Importantly, MCI remains a significant diagnostic challenge and is notoriously underdiagnosed, with literature indicating that up to 92% of cases are overlooked in clinical practice^20,21^. Our findings align with previous research indicating that the QDRS is highly effective at identifying functional decline, but less sensitive to the subtle changes associated with the early stages of cognitive impairment and the transition from normal cognition to MCI. Integrating the informant-based QDRS with sensitive objective patient-derived measures, such as the MoCA, may enhance the detection of early-stage cognitive decline by capturing both functional status and subtle cognitive deficits. 

 Notably, the QDRS demonstrated robust psychometric performance across both primary and secondary care settings in this study. It exhibited high internal consistency, with a Cronbach’s alpha of 0.94, indicating excellent homogeneity among its items. Concurrent validity was supported by moderate to strong correlations, with a Spearman’s rho of 0.82, reflecting a robust association with established clinical measures in accordance with the existing literature^10,11^. Furthermore, staging agreement analyses yielded a Kappa coefficient of 0.64, indicating substantial agreement, which supports the QDRS ability to accurately classify stages of cognitive impairment and these results are consistent with previous studies^22^. These findings are consistent with previous validation studies of the QDRS, which also reported high internal consistency and substantial agreement with clinical staging instruments. Furthermore, our study breaks new ground by validating the QDRS across the cognitive continuum, the findings confirm the instrument as an exceptionally reliable tool for the primary care system, where rapid and accurate screening is most critical. 

 Although the findings were robust overall, certain limitations must be considered. First, in contrast to the original validation study, the present sample did not allow for reliable discrimination among specific dementia severity stages (CDR 1, 2, and 3). Additionally, validation of the QDRS behavioral domain could not be undertaken, as standardized behavioral assessments were not consistently available across all cohorts included in this study. Participants in this study presented higher levels of education for older adults in Brazil^23^, and these aspects may have increased the apparent diagnostic performance of the instruments. Further research with larger, education-stratified samples and standardized behavioral scales is warranted, including with different dementia syndromes to ensure replicability across different etiologies of cognitive decline. It is important to note that the current validation was conducted in research settings, and then scores were evaluated by clinicians specialized in memory evaluation. Considering that the QDRS is an informant-based questionnaire, it is important to acknowledge that certain biases may arise. Strategies should be implemented to mitigate these effects, particularly through clear instructions regarding perspective — explicitly specifying whether the informant should respond from their own perspective or from the patient’s perspective. Lack of clarity in this regard is common and may compromise response consistency^24,25^. Additionally, it is important to recognize that the informant’s demographic and relational characteristics may influence their responses, and further studies should incorporate complete informant’s data. Moreover, the QDRS demonstrated limited accuracy to identify MCI, potentially associated with the absence of a comprehensive neuropsychological battery. Therefore, further studies are required to ensure that its diagnostic performance and efficacy remain consistent in real-world settings. 

 In conclusion, the Brazilian Portuguese version of the QDRS constitutes a highly robust and top-tier diagnostic instrument, demonstrating extraordinary accuracy in distinguishing CU individuals and those with MCI from dementia across primary and secondary care. Ultimately, the QDRS is a transformative asset for the Brazilian healthcare landscape. Given the continental reach of the public health system (SUS), it provides a scalable, high-precision staging dementia across routine clinical care, large-scale epidemiological surveys, and the rigorous demands of biomarker-driven prevention trials. 

## Data Availability

The data that support the findings of this study are available from the corresponding author upon reasonable request. Due to privacy and ethical restrictions, individual participant data cannot be shared publicly.
