# Supplementary material for: Validation of the Quick Dementia Rating System (QDRS): diagnostic properties and cutoff values
Source: Dement Neuropsychol. 2026 Aug 24;20:e20260520. doi: 10.1590/1980-5764-DN-2026-0520 (PMC13506179; doi:10.1590/1980-5764-DN-2026-0520)
Supplement: Supplementary file 1 [file 1980-5764-dn-20-e20260520-md01.docx]

**Supplementary Material**

**Translation and Back Translation procedures**

The cross-cultural adaptation of the Quick Dementia Rating System (QDRS) into Brazilian Portuguese was performed following internationally recognized guidelines to ensure idiomatic, semantic, and conceptual equivalence between the original English instrument and the target version. The process was divided into the following four stages:

1. The initial phase involved a thorough review of the original QDRS items to identify potential cultural nuances or linguistic constructs that might not have a direct equivalent in the Brazilian context. Wording transformations were applied to ensure that the clinical descriptors remained relevant to the Brazilian socioeconomic and educational landscape while maintaining the original's intent to measure cognitive and functional decline.
2. Two independent native Brazilian Portuguese speakers, both fluent in English, translated the original instrument. Translator 1 was aware of the clinical concepts and the construct being measured (providing a clinical perspective). Translator 2 was not informed of the clinical objectives (providing a "naive" linguistic perspective to ensure common language usage). Both versions were compared, and a synthesized version was produced to resolve minor discrepancies in terminology.
3. To ensure the integrity of the translated content, the synthesized Portuguese version was subjected to back-translation. This was performed by a native English speaker fluent in Portuguese who is a professional translator and had no prior knowledge of the original QDRS. This step was crucial to verify that the translated items did not deviate from the original meaning or introduce unintended biases.
4. An expert panel reviewed the original version, the forward translations, and the back-translation. This committee ensured that the final Brazilian Portuguese version achieved cross-cultural equivalence across four domains: Semantic equivalence; Idiomatic equivalence; Experiential equivalence and Conceptual equivalence.

After the 4-stage cross-validation translation and back translation procedures were finalized, we conducted the diagnostic properties calculation described in the **Method section.**

**Table S1.** Quick Dementia Rating System - informant version.

| As seguintes descrições caracterizam as mudanças nas habilidades cognitivas e funcionais do paciente. Você deve comparar o paciente agora e como ele costumava ser - a principal característica é a **mudança.** Escolha **uma resposta** para cada categoria que melhor descreve o paciente - PRESTE ATENÇÃO, nem todas as descrições precisam corresponder completamente para escolher uma resposta. | | |
| --- | --- | --- |
| **Domínio** | **Ponto** | **Resposta** |
| **1. Memória e lembrança** | 0 | Sem perda de memória evidente, ou esquecimento inconsistente, que não interfere na funcionalidade de atividades cotidianas. |
|  | 0.5 | Esquecimento leve consistente ou lembrança parcial de eventos que podem interferir com atividades de vida diária; repete perguntas/afirmações, perde itens, esquece compromissos. |
|  | 1 | Perda de memória leve a moderada; mais notável para eventos recentes; interfere com atividade de vida diária. |
|  | 2 | Perda de memória de moderada a grave; lembra somente informações altamente aprendidas; novas informações são rapidamente esquecidas. |
|  | 3 | Perda grave de memória, quase impossível lembrar novas informações; memória de longo-prazo pode estar afetada. |
| **2. Orientação** | 0 | Quase sempre está completamente orientado das pessoas ao seu redor, do local e do tempo. |
|  | 0.5 | Leve dificuldade em ter noção do tempo; pode esquecer o dia ou data mais frequentemente que no passado. |
|  | 1 | Leve a moderada dificuldade em manter noção de tempo e sequência de eventos; esquece mês ou ano; orientado em locais familiares, mas se confunde em áreas novas; fica perdido ou anda sem rumo. |
|  | 2 | Moderada a grave dificuldade, geralmente desorientado em tempo e espaço (familiar ou não-familiar); frequentemente menciona o passado. |
|  | 3 | Somente orientado com seu nome, embora possa reconhecer familiares. |
| **3. Tomada de decisões e resolução de problemas** | 0 | Soluciona problemas diários sem dificuldade; lida bem com negócios pessoais e financeiros; habilidades de tomada de decisão consistente com a performance no passado. |
|  | 0.5 | Soluciona problemas com leve dificuldade ou leva mais tempo para solucioná-las; problemas com conceitos abstratos; decisões ainda razoáveis. |
|  | 1 | Moderada dificuldade em lidar com problemas e tomar decisões; delega muitas decisões para outros; julgamento social e comportamento podem estar levemente alterados; perda de clareza. |
|  | 2 | Grave prejuízo em lidar com problemas, realiza apenas decisões pessoais simples; julgamento social e comportamento frequentemente prejudicados; falta clareza. |
|  | 3 | Incapaz de tomar decisões ou resolver problemas; outros tomam quase todas as decisões pelo paciente. |
| **4. Atividades fora de casa** | 0 | Independência funcional a nível profissional, nas compras, na comunidade, nas atividades comunitárias e religiosas, no voluntariado ou nos grupos sociais. |
|  | 0.5 | Leve prejuízo nestas atividades quando comparadas a performance prévia; leve mudança em conduzir veículos; ainda consegue manejar situações de emergência. |
|  | 1 | Incapaz de ser funcional e independente mas ainda participa e se engaja em atividades; parece "normal" aos olhos de outros; mudanças importantes de direção de veículos; questionável habilidade pra manejar situações de emergências. |
|  | 2 | Não intenta ser funcional e independente fora de casa; aparenta estar bem o suficiente para ir em atividades fora da casa da família, mas geralmente precisa estar acompanhado. |
|  | 3 | Sem independência de funcionalidade ou de atividades; parece muito doente para atividades fora de casa. |
| **5. Função em casa e lazer** | 0 | Tarefas de casa, atividades de lazer e interesses pessoais são bem mantidos quando comparado com o passado. |
|  | 0.5 | Leve prejuízo ou interesse nessas atividades; problemas em operar aparelhos (especialmente equipamentos recém adquiridos). |
|  | 1 | Leve mas consistente prejuízo nas funções de casa e atividades de lazer; faz tarefas com mais dificuldade ou abandona; desistiu de atividades de lazer mais complicadas. |
|  | 2 | Somente tarefas simples preservadas; interesse muito restrito nas atividades de lazer, os quais são mal mantidos. |
|  | 3 | Sem função significativa nas tarefas de casa ou em atividades de lazer prévios. |
| **6. Cuidado e higiene pessoal** | 0 | Completa capacidade de auto-cuidado (vestir, pentear o cabelo, fazer a barba, lavar as mãos, tomar banho, usar a privada). |
|  | 0.5 | Pequenas mudanças nas habilidades e atenção para essas atividades. |
|  | 1 | Precisa estímulo para completar essas atividades, mas faz independentemente. |
|  | 2 | Precisa de ajuda para se vestir, realizar higiene pessoal, cuidar de itens pessoais; ocasionalmente incontinente. |
|  | 3 | Requer ajuda significativa para higiene e cuidado pessoal; incontinência frequente. |
| **7. Mudanças de personalidade e comportamento** | 0 | Comportamento socialmente adequado em publico e privado. Sem mudanças de personalidade. |
|  | 0.5 | Alterações muito leves ou questionáveis no comportamento, personalidade, controle emocional ou escolhas adequadas. |
|  | 1 | Mudança leve no comportamento ou na personalidade. |
|  | 2 | Mudança moderada de personalidade ou comportamento afeta interação com outras pessoas; pode ser evitado por amigos, vizinhos ou parentes distantes. |
|  | 3 | Mudança grave no comportamento ou personalidade; interações com outras pessoas é frequentemente desagradável ou evitada. |
| **8. Linguagem e comunicação** | 0 | Sem dificuldade de linguagem ou busca eventual de palavras; lê e escreve como fazia no passado. |
|  | 0.5 | Dificuldade leve e consistente para encontrar palavras, usa termos descritivos da palavra ou leva mais tempo para se explicar, leve problema de compreensão, conversação reduzida; pode afetar leitura ou escrita. |
|  | 1 | Moderada dificuldade para encontrar palavras ao falar, não nomeia objetos, redução pronunciada na fluência do discurso; redução na compreensão, conversação, leitura e/ou escrita. |
|  | 2 | Prejuízo moderado a grave de produção de discurso e compreensão; dificuldade em se expressar; habilidade limitada para de ler e escrever. |
|  | 3 | Prejuízo grave na linguagem e comunicação; discurso pouco compreensível ou incompreensível. |
| **9. Humor** | 0 | Sem mudança de humor, de interesses e de motivação. |
|  | 0.5 | Ocasional tristeza, depressão, ansiedade, nervosismo, ou perda de interesse/motivação. |
|  | 1 | Episódios leves diários de tristeza, depressão, ansiedade, nervosismo, perda de interesses/motivação. |
|  | 2 | Episódios moderados de tristeza, depressão, ansiedade, nervosismo, perda de interesses/motivação. |
|  | 3 | Episódios graves de tristeza, depressão, ansiedade, nervosismo, perda de interesses/motivação. |
| **10. Atenção e concentração** | 0 | Atenção, concentração e interação com o ambiente e seus arredores estão normais. |
|  | 0.5 | Leve problemas de atenção, concentração, interação com o ambiente e arredores, pode parecer sonolento durante o dia. |
|  | 1 | Problemas moderados com atenção e concentração, pode apresentar olhar fixado em um ponto ou ficar de olhos fechados; aumento de sonolência diurna. |
|  | 2 | Dorme uma porção significativa do dia, não presta atenção ao ambiente; quando há uma conversa, pode dizer coisas ilógicas ou inconsistentes com o assunto. |
|  | 3 | Habilidade limitada ou inexistente em prestar atenção em ambientes externos e arredores. |

| **Total cognitivo**  (somar 1,2,3,8) |  | **Total comportamental**  (somar 4,5,6,7,9,10) |  | **Total**  **QDRS** |  |
| --- | --- | --- | --- | --- | --- |

**Table S2.** Demographic and clinical characteristics by clinical diagnostic groups according to different cohorts.

|  | **ELVE cohort** | | | **PORTO-AD cohort** | | |
| --- | --- | --- | --- | --- | --- | --- |
|  | **CU**  (n = 14) | **MCI**  (n = 34) | **Dementia**  (n = 27) | **CU**  (n = 45) | **MCI**  (n = 29) | **Dementia**  (n = 31) |
| Age, years, Mean (SD) | 74.1 (4.7) | 76.5 (6.2) | 77.6 (5.2) | 58.4 (8.6) | 62.9 (9.2) | 74.4 (7.6) |
| Education, years, Mean (SD) | 8.9 (5.4) | 6.7 (4.9) | 7.6 (5.2) | 15.5 (6.9) | 11.7 (5.2) | 12.8 (7.8) |
| Female Sex, n (%) | 14 (100%) | 17 (50%) | 17 (63%) | 34 (76%) | 11 (38%) | 20 (65%) |
| Race, n (%) |  |  |  |  |  |  |
| White | 14 (100%) | 34 (100%) | 26 (96%) | 36 (80%) | 26 (90%) | 30 (97%) |
| Brown | 0 (0%) | 0 (0%) | 1 (3.7%) | 2 (4.4%) | 1 (3.4%) | 0 (0%) |
| MMSE Total Score, Mean (SD) | 27.0 (2.5) | 24.4 (3.3) | 16.9 (5.0) | 7 (16%) | 2 (6.9%) | 1 (3.2%) |
| MoCA Total Score, Mean (SD) | 23.4 (4.0) | 19.8 (4.0) | 11.4 (5.5) | 26.9 (2.4) | 24.3 (3.4) | 19.1 (5.4) |
| Verbal Fluency (words), Mean (SD) | 9.3 (4.2) | 8.0 (3.5) | 5.7 (3.5) | 24.4 (3.5) | 20.0 (4.1) | 14.8 (4.7) |
| QDRS Total Score, Mean (SD) | 0.1 (0.2) | 2.6 (4.1) | 13.1 (7.0) | 13.2 (4.7) | 11.6 (4.9) | 7.6 (3.8) |
| QDRS Cognitive Subscale, Mean (SD) | 0.1 (0.2) | 1.2 (1.9) | 6.2 (3.0) | 0.9 (1.8) | 3.3 (3.5) | 9.5 (5.5) |
| QDRS Behavioral Subscale, Mean (SD) | 0.0 (0.0) | 1.3 (2.3) | 6.8 (4.5) | 0.2 (0.6) | 1.0 (1.1) | 4.0 (2.5) |
| CDR Global Score, n (%) |  |  |  | 0.7 (1.3) | 2.3 (2.6) | 5.5 (3.3) |
| 0 | 10 (71%) | 7 (21%) | 0 (0%) |  |  |  |
| 0.5 | 4 (29%) | 25 (74%) | 3 (11%) | 44 (98%) | 10 (34%) | 0 (0%) |
| 1 | 0 (0%) | 2 (5.9%) | 9 (33%) | 1 (2.2%) | 18 (62%) | 2 (6.5%) |
| 2 | 0 (0%) | 0 (0%) | 13 (48%) | 0 (0%) | 1 (3.4%) | 22 (71%) |
| 3 | 0 (0%) | 0 (0%) | 2 (7.4%) | 0 (0%) | 0 (0%) | 7 (23%) |
| CDR Sum of Boxes, Mean (SD) | 0.2 (0.4) | 1.1 (1.5) | 8.7 (4.9) | 0.0 (0.1) | 1.0 (0.9) | 5.5 (2.9) |

Abbreviations: CU, Cognitively Unimpaired; MCI, Mild Cognitive Impairment; MMSE, Mini-Mental State Examination; MoCA, Montreal Cognitive Assessment; QDRS, Quick Dementia Rating System; CDR, Clinical Dementia Rating.

**Figure S1.** Spearman correlation matrix among demographic variables and cognitive, functional and global severity measures. Blue indicates positive and orange negative correlations, with color intensity reflecting strength.

Abbreviations: QDRS Cogn, Quick Dementia Rating System – Cognitive subscale; QDRS Behav, Quick Dementia Rating System - Behavioral subscale; QDRS Total, Quick Dementia Rating System - Total score; CDR Total, Clinical Dementia Rating - Global score; CDR-SOB, Clinical Dementia Rating - Sum of Boxes; CDT, Clock Drawing Test; MMSE, Mini-Mental State Examination; MoCA, Montreal Cognitive Assessment; Verbal Fluency, Verbal Fluency Test; Age, participant age in years; Education, years of formal education.

**Figure S2.** Diagnostic accuracy of the QDRS in a community-dwelling subsample (ELVE). Receiver Operating Characteristic (ROC) curves demonstrating the discriminative ability of the QDRS total score against (a) clinical diagnostic groups and (b) Clinical Dementia Rating (CDR) stages. Curves illustrate the test's ability to distinguish between different clinical scenarios.

**Figure S3.** Diagnostic accuracy of the QDRS in a secondary care subsample (PORTO-AD). Receiver Operating Characteristic (ROC) curves demonstrating the discriminative ability of the QDRS total score against (a) clinical diagnostic groups and (b) Clinical Dementia Rating (CDR) stages. Curves illustrate the test's ability to distinguish between different clinical scenarios.

**Figure S4.** Flowchart of study inclusion, for both ELVE and PORTO-AD cohorts.
